# Prognostic Significance of Serum PSA Level and Telomerase, VEGF and GLUT-1 Protein Expression for the Biochemical Recurrence in Prostate Cancer Patients after Radical Prostatectomy

**DOI:** 10.1007/s12253-019-00659-4

**Published:** 2019-04-15

**Authors:** Anna Gasinska, Janusz Jaszczynski, Urszula Rychlik, Elżbieta Łuczynska, Marek Pogodzinski, Mikolaj Palaczynski

**Affiliations:** 1 Department of Tumour Pathology, Oncology Center, Maria Sklodowska - Curie Institute, Cracow Branch, Garncarska 11, 31-115, Cracow, Poland; 2 Department of Surgery, Oncology Center, Maria Sklodowska - Curie Institute, Cracow Branch, Cracow, Poland; 3 Department of Clinical Biochemistry, Oncology Center, Maria Sklodowska-Curie Institute, Cracow Branch, Cracow, Poland; 4 Department of Radiology, Oncology Center, Maria Sklodowska-Curie Institute, Cracow Branch, Cracow, Poland

**Keywords:** Prostatic carcinoma, Biochemical failure, PSA, GLUT-1, VEGF, hTERT

## Abstract

The aim of the study was to evaluate prognosis for biochemical recurrence (BR) by analysing the pathological and biological characteristics of prostate cancer (PCa) after radical prostatectomy (RP). There were 130 men with clinically localized PCa in whom pretreatment serum PSA level and Ki-67, prostate specific membrane antigen (PSMA), glucose transporter-1 (GLUT-1), vascular endothelial growth factor (VEGF), microvessel density (MVD) and human telomerase reverse transcriptase (hTERT) proteins expression, based on number of immunohistochemically positive cells (labelling index), were retrospectively studied. In order to assess the prognostic significance of analysed variables in univariate and multivariate Cox analysis, patients were dichotomized based on cut-off points chosen by receiver operating characteristic (ROC) curves. There were 83 males (63.8%) at pT stage 1–2 and 47 (36.1%) at pT stage 3–4, respectively, with median (range) age of 62.8 years (49–77), and median follow-up of 78.5 months (12–148). In 42 (32.3%) men BR was found. In univariate analysis, tumour biological features: PSA ≤ 8 ng/mL (*p* = 0.006), Ki-67LI ≤ 12.7% (*p* = 0.015), VEGFLI>11.0% (*p* = 0.030), and hTERTLI>6.7% (*p* = 0.016), but not clinicopathological parameters, appeared to be positive prognosticators for BRFS. In the Cox analysis, Ki-67 lost its significance, and clinicopathological parameters appeared to be nonsignificant. The independent negative prognostic factors for BRFS were: PSA > 8.0 ng/mL, (Hazard ratio = 2.75*, p* = 0.003), GLUT-1 > 19.1% (HR = 2.1, *p* = 0.032), VEGF≤11.0% (HR = 1, *p* = 0.024) and hTERT≤6.7% (HR = 1, *p* = 0.017). High PSA level, and GLUT-1 expression and lower VEGF and nuclear hTERT expression may indicate the great role of hypoxia in BR induction in PCa.

## Introduction

Prostate cancer (PCa), the most common malignancy in males, is characterized by intratumoral heterogeneity and a viable clinical course. Traditionally, prostate cancer diagnosis is based on prostate specific antigen (PSA) determination and clinicopathological factors (histology, tumour size, Gleason scores and clinical staging). However, there are remarkable differences in the biological behavior of prostate cancers classified as the same grade and stage, as clinical prognostic groupings for localized PCa are imprecise. Therefore, reliable distinction between indolent and aggressive PCa prior to treatment implementation is not achievable [1]. In low-advanced PCa, both radiotherapy (RT) and radical prostatectomy (RP) are primary treatment modalities. However, RP remains the gold standard for curative treatment of PCa because it significantly reduces mortality, the risk of local progression and the onset of distant metastasis [2].

Prostate-specific antigen (PSA) has been generally used for screening of PCa, post diagnostic PSA surveillance or monitoring following treatment. Cancer progression defined by elevated PSA level (two consecutive PSA levels ≥ 0.2 ng/mL), otherwise known as biochemical recurrence (BR), is almost always the earliest sign of recurrent PCa and can predate either clinical or radiographic evidence of disease by months to years following RP or radiotherapy [3, 4]. However, qualification to treatment of individual patient is imprecise and 30–50% of patients have biochemical relapse within 10-years after RP or image-guided radiotherapy [5, 6], which would never progress to clinical metastasis. Therefore, it is now considered, that PSA has limited diagnostic and prognostic value and a low specificity and sensitivity resulting in frequent misdiagnosis [3]. Furthermore, PSA test has limitations including lack of standardization of screening as serum levels exhibit relatively wide biological variations and PSA levels do not accurately predict disease aggressiveness (different PSA cut-off points and frequency of screening schedules) [1, 3]. Also the test has potential harms for prostate cancer screening and patients’ follow-up (overdiagnosis - false positive, complications of unnecessary biopsy) or overtreatment (surgery, radiation treatment) [3, 4].

Therefore, the availability of better prognostic biomarkers may also greatly aid the treatment decision process for PCa patients. Early recognition based on biological markers could be helpful not only in identification of biological differences in benign and malignant lesions, offering further help in precise indication for more aggressive post-operative treatment (chemo/radiotherapy) but also for assessment of BR risk. Immunohistochemical expressions of many molecular tissue markers in PCa have been studied, such as: cyclooxygenase 2, P53, Ki-67, BCL2 and microvessel density [7–9], but their actual clinical usefulness has not yet been conclusively validated and approved for routine assessment [10]. In another study, BR was associated with tumour aggressive features in the DNA-based and RNA-based signatures to measure genomic instability [11]. Recently, radiomics or computer-extracted texture features derived from magnetic resonance imaging (MRI) [12] or metabolomics [13] have been shown to help quantitatively characterize PCa and predict BR or malignancy of PCa .

Earlier, we checked, based of pretreatment PSA level and immunohistochemical analysis of 6 proteins, if it is possible to select, before RP, more aggressive tumours for more aggressive treatment. We showed that increase of pathological tumour volume and tumour grade was associated with statistically significant increase in serum PSA, Ki-67 [14] and prostate specific membrane antigen (PSMA) expression [15], however, for telomerase enzyme activity (human telomerase reverse transcriptase, a catalytic subunit of telomerase, hTERT) the relation was opposite, indicating extranuclear telomere activity independent of telomere lengthening, and suggesting that it cannot be considered as a marker of malignancy [15]. However, significantly higher vascular endothelial growth factor (VEGF) was observed in pT3 and pT4 than in pT1 stage [16] indicating induction of angiogenesis and endothelial cell growth, as it is considered to be hypoxia-inducible pro-angiogenic protein [17]. Microvessel density (MVD/CD34) and glucose transporter −1through the cell membrane (GLUT-1) were not significantly correlated with tumour volume and grade [14, 16]. Therefore, the aim of the present project is to check, in the same patient cohort, the possibility of showing prognostic significance of pretreatment PSA level, Ki-67LI, MVD, VEGFLI, GLUT-1LI, PSMALI and hTERT activity in PCa for the frequency of biochemical relapses and patients’ biochemical recurrence-free survival (BRFS). In order to avoid predetermined cutoff point for biological variables, receiver operating characteristic (ROC) curve analysis was applied.

## Material and Methods

### Patients and Samples

The studied population consisted of a retrospective cohort of 130 PCa patients who had RP surgery between 2007 -2011and in whom tumour biopsy was taken during curative surgery. Tumours were classified according to clinical T category (cTNM) and pathological (pTNM) stages, according to the American Joint Committee on Cancer (AJCC) guidelines and Gleason score. The protocol was approved by the Ethical Committee of the Centre of Oncology, and each patient submitted written consent.

### Immunohistochemical Analysis and Scoring

Protein expression was evaluated immunohistochemically on histological specimens, using the suitable antibody and BrightVision visualization system (ImmunoLogic). For Ki-67 visualization we used a mouse-antiKi-67 monoclonal antibody (clone MIB-1, DAKO, 1:75), for GLUT-1 a rabbit monoclonal antibody (Millipore, 1:300), for CD34 a mouse anti-human monoclonal antibody (DAKO, 1:200), for VEGF a mouse anti-VEGF monoclonal antibody (DAKO, 1:25), for hTERT a rabbit polyclonal antibody to human telomerase (Novus Lab. Biologicals, Littleton, USA), (1:300) and for PSMA a mouse anti-human prostate specific membrane antigen (Novocastra, Newcastle, United Kingdom)(1:200), diluted in TRIS-buffered saline (pH = 7.4), as described earlier [14–16]. Proteins expression was presented as the number of positively staining cells (labelling index, LI) of nuclear (Ki-67, hTERT), membrane (PSMA, CD34), membrane/cytoplasmic (GLUT-1) and cytoplasmic (VEGF) staining and MVD (CD34 immunoreactivity) as a mean vessel count per 1 mm^2^ of tumour volume. Slides were evaluated by two investigators who were unaware of the clinicopathological variables.

### Evaluation of Follow-Up

After surgery, patients were followed with PSA measurements at 3 and 6 months, then every 6 months for 2 years, and thereafter yearly or until biochemical recurrence (BR). No patients received adjuvant hormonal or radiation therapy before BR was confirmed. BRFS was the time calculated from the date of surgery to BR, which was defined as detectable serum PSA (greater than 0.2 ng/mL), as documented by repeated PSA measurements.

### Statistical Analysis

Statistical analysis was performed with STATISTICA vs 12 (StatSoft Inc. Tulusa, OK, USA). To determine mean values for variables and standard errors of means (SE) we used the descriptive statistics. One-way ANOVA test or Student’s t test were applied to test intergroup differences in the mean values. The correlations between proteins expression were tested with Pearson correlation and between proteins expression and other variables with Spearman rank test. Associations between investigated categorical parameters and clinicopathological variables were evaluated by Pearson’s Chi^2^ test. For sensitivity and specificity of each marker receiver, curve analyses (ROC curves) were performed to determine the area under the curve (AUC) as a measure of diagnostic accuracy. For BRFS analysis patients were stratified into two groups based on cutoff points for each variable optimized by ROC curve.

### Selection of Cutoff Score

ROC curve analysis was utilized to determine cutoff value for separating tumors with abnormal proteins expression from tumors with normal expression by using the 0. 1-criterion. The sensitivity and specificity for each outcome under study was plotted, thus generating various ROC curves with areas under curve (AUCs). The score was selected as the cutoff value, which was closest to the point with both maximum sensitivity and specificity. Tumors designated as “normal expression” were those with scores below or equal to the cutoff value, while tumors which ‘overexpression” were those with scores above the value. In order to perform ROC curve analysis, the clinicopathologic features were dichotomized: age (≤61.0 or > 61.0 years), AJCC tumor grade (G1 or G2/3), TNM stage (T1–2 or T3–4), pTNM (pT1/2 or pT3/4). Survival was estimated using the Kaplan-Meier method and tested by the log-rank test. A univariate and multivariate Cox proportional model was conducted to further explore the relationship between proteins expression and the BRFS. In multivariate analysis, clinical and biological variables were first tested separately and finally all variables together. Statistical significance was considered at *p* value of 0.05.

## Results

A total of 130 patients with a median age of 62.0 years were eligible for this study. (Table 1). During a median follow-up of 78.5 (range 12.0–148.0) months, 42 (32.3%) patients developed BR within 8–86 month (median 35) months after RP. The clinicopathological characteristics of the patients are summarized in Table 1. There was no statistically significant difference between age and clinicopathological variables between patients with and without BF, except Gleason score; a higher number of more anaplastic tumours had BF (Table 1).

**Table 1:** Clinicopathological characteristics of 130 patients with PCa treated with radical prostatectomy (comparison between biochemical recurrence (BR) - positive versus BR-negative subgroups)

Characteristics	Total *N* = 130 (%)	BR-negative* *N* = 88	BR-positive** *N* = 42	*p* value
Median age, years (range)	(130) 62.0	62.0	64.0	0.368
	(49–77)	(50.0–75.0)	(49.0–77.0)	
cT stage				
T1	11 (8.5)	8 (9.1)	3 (7.1)	0.554
T2A	14 (10.8)	10 (11.4)	4 (9.5)	
T2B	57 (43.8)	38 (43.2)	19 (45.2)	
T3	37 (28.5)	26 (29.5)	11 (26.2)	
T4	10 (7.7)	5 (5.7)	5 (11.9)	
Tx	1 (0.8)	1	0	
pT stage				
pT1	12 (9.2)	9 (10.2)	3 (7.1)	0.606
pT2	71 (54.6)	47 (53.4)	24 (57.1)	
pT3	38 (29.2)	28 (31.8)	10 (23.8)	
pT4	9 (6.9)	4 (4.5)	5 (11.9)	
Histological grade AJCC				
1	69 (53.1)	51 (57.9)	18 (42.8)	0.087
2	49 (37.7)	30 (34.1)	19 (45.2)	
3	12 (9.2)	7 (7.9)	5 (11.9)	
Gleason Score				
≤ 6	67 (51.5)	51 (57.3)	17 (40.5)	0.047
7	50 (38.5)	31 (34.8)	19 (45.2)	
≥ 8	13 (10.0)	7 (7.9)	6 (14.3)	

* PSA <= 0.2 ng/mL, ** PSA > 0.2 ng/mL

The mean values of biomarkers were given in Table 2. There was no statistically significant difference in the mean values of biomarkers in patients who were PSA failure positive and negative (Table 2).

**Table 2:** Tumour biological characteristics and pretreatment PSA levels in patients with biochemical recurrence (BR-positive) and without BR (BR- negative)

Characteristics	Total *N* = 130 Mean ± SE	BR-negative *N* = 88 Mean ± SE	BR-positive *N* = 42 Mean ± SE	*p* value*
PSA ng/mL	9.9 ± 0.5	9.4 ± 0.6	10.8 ± 0.7	0.195
Ki-67 (%)	8.1 ± 0.6	7.3 ± 0.6	9.7 ± 1.3	0.092
GLUT-1(%)	30.4 ± 2.1	29.6 ± 2.4	31.9 ± 3.8	0.603
VEGF (%)	15.1 ± 1.4	16.1 ± 1.8	12.8 ± 2.3	0.281
MVD/mm^2^	97.1 ± 2.5	99.1 ± 3.1	92.8 ± 3.9	0.234
PSMA (%)	44.6 ± 2.0	44.6 ± 2.2	44.8 ± 3.9	0.950
hTERT (%)	18.3 ± 1.6	20.3 ± 2.0	14.3 ± 2.3	0.075

*Difference between BR- positive and negative subgroups (t-test)

### Correlation between Clinical and Biological Variables

The expression level of PSA (*p* = 0.030), Ki-67 (*p* = 0.024), PSMA (*p* < 0.001), GLUT-1 (*p* = 0.037) were positively and hTERT (*p* = 0.033) negatively correlated with the Gleason score. Only PSA (*p* = 0.007) and PSMA (p < 0.001) were positively correlated with cT and pT stage (p < 0.001). VEGF expression was not linked to any clinicopathological variables. Further correlation analysis showed that in our cohort, only the expression of PSA was positively correlated with Ki-67 (*p* = 0.014). There were no significant correlations between the expressions of other markers .

### Selection of Cutoff Points

According to the ROC curve, the cutoff points for the analysed biomarkers: PSA, Ki-67, CD34, GLUT-1, VEGF, PSMA, hTERT were defined for 8 ng/mL, 12.7%, 82.5 microvessels/mm^2^, 19.1%, 11.0%, 49.3% and 6.7%, respectively.

The ROC curve for the value of the following biomarkers in prediction of postoperative biochemical recurrence of PCa showed the AUC to be 0.635 (95% CI: 0.534–0.736), with a sensitivity of 73.2% and a specificity of 56.2% for PSA, 0.564 (95% CI: 0.451–0.679), with a sensitivity of 29.3% and a specificity of 89.9x % for Ki-67, 0.450 (95% CI: 0.343–0.556), with a sensitivity of 61.0% and a specificity of 24.7% for CD34, 0.512 (95% CI: 0.403–0.620), with a sensitivity of 70.7% and a specificity of 44.9% for GLUT-1, 0.505 (95% CI: 0.394–0.618), with a sensitivity of 52.4% and a specificity of 64.1% for PSMA, 0.536 (95% CI: 0.432–0.640), with a sensitivity of 63.4% and a specificity of 52.8% for VEGF, 0.615 (95% CI: 0.512–0.718), with a sensitivity of 73.0% and a specificity of 46.3% for telomerase.

### Univariate and Multivariate Analysis

**Fig. 1:**
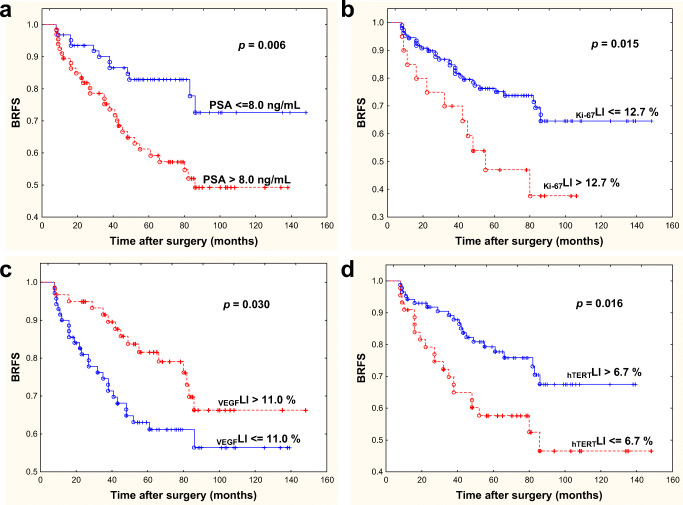
Prognostic significance of PSA (a), Ki-67 (b), VEGF (c), hTERT (d), for biochemical recurrence-free survival (BRFS) in prostate cancer patients treated with radical prostatectomy (*p* value from log-rank test)

Kaplan-Meier analysis showed the actuarial patients’10-year BRFS as 60%. In log-rank test PSA ≤8 ng/mL (*p* = 0.006), Ki-67LI ≤ 12.7% (*p* = 0.015), hTERTLI>6.7% (*p* = 0.016) and VEGFLI>11.0% (*p* = 0.030) were positive statistically significant prognosticators for BRFS (Fig. 1). An univariate Cox proportional model was conducted to further explore the relationship between proteins expression and BF. In the analysis, PSA, Ki-67, VEGF and hTERT appeared to be important prognostic factors for BRFS (Table 3). When only clinicopathological variables were included in Cox analysis, positive prognosticators for BRFS were: cT1–2 (*p* = 0.024), pT 1–2 (*p* = 0.018), and Gleason score < 7 (*p* = 0.043). However, when PSA was added, only PSA became independent prognostic factor (*p* = 0.005). When all clinical and biological parameters were analysed together, none of the clinical and pathological features of PCa was shown to have greater impact on patients’ BRFS than tumour biological features. High pretreatment levels of serum PSA (> 8 ng/mL), GLUT-1LI (>19.1%) and lower hTERT (≤6.7%) and VEGF (≤11.0%) expression were significant predictors for shorter time to BR on multivariate analysis (Table 3).

**Table 3:** Cox proportional hazards analysis of the biological and clinicopathological features of prostate cancer predicting biochemical recurrence-free survival in men with localized PCa

Variable	Univariate analysis			Multivariate analysis		
	RR	95% CI	*p* value*	RR	95% CI	*p* value
Age (years)						
≤ 61.0	1.00	Reference				
> 61.0	1.20	0.65–2.20	0.550			
AJCC Grade						
1	1.00	Reference				
2–3	1.78	0.96–3.32	0.070			
Clinical stage						
cT1–2	1.00	Reference	0.929			
cT3–4	1.03	0.55–1.92				
Pathological stage						
pT1–2	1.00	Reference				
pT3–4	0.91	0.48–1.71	0.765			
PSA						
≤ 8.0 ng/mL	1.00	Reference		1.00	Reference	
> 8.0 ng/mL	2.53	1.29–4.96	0.006	2.75	1.40–5.43	0.003
Ki-67LI						
≤ 12.7%	1.00	Reference				
>12.7%	2.35	1.17–4.69	0.015			
GLUT-1LI						
≤ 19.1%	1.00	Reference		1.00	Reference	
> 19.1%	1.63	0.83–3.13	0.143	2.10	1.1–4.1	0.032
VEGF						
≤ 11.0%	1.00	Reference		1.00	Reference	
> 11.0%	0.53	0.28–1.00	0.030	0.46	0.24–0.90	0.024
MVD						
≤ 82.5/mm^2^	1.00	Reference				
> 82.5/mm^2^	0.68	0.36–1.27	0.225			
PSMA						
≤ 49.3%	1.00	Reference				
> 49.3%	1.30	0.69–2.34	0.428			
hTERT LI						
≤ 6.7%	1.00	Reference		1.00	Reference	
> 6.7%	0.47	0.25–0.87	0.016	0.47	0.25–0.87	0.017

*Abbreviations*: *RR* risk ratio, *CI* confidence interval

## Discussion

We analysed the prognostic value of pretreatment serum PSA level and expression of 6 proteins for BRFS of low and intermediate risk PCa patients after RP. We have shown that none of the analyzed clinical and pathological features of PCa was shown to have a greater impact on patients’ BRFS than tumour biological features, which is in agreement with recently published studies showing greater significance of biomarkers than clinic-pathological features in risk assessment for BR after RP [6, 11, 18].

We showed that high PSA levels (> 8 ng /mL) and tumour hypoxia (GLUT-1 > 19.1%) or lower nuclear hTERTLI (≤6.7%) and VEGFLI (≤11.0%) expression were negative prognostic factors for BRFS. In the multivariate analysis the PSA level > 8.0 ng/mL and higher fraction of hypoxic cells (GLUT-1 > 19.1%) doubled the risk (HR = 2), while higher VEGF and nuclear hTERT expression reduced by half (HR = 0.5) the risk of biochemical recurrence in PCa patients after prostatectomy. All these indicate the great role of hypoxia in induction of BR in PCa.

In our cohort, the overall PSA recurrence rate was 32.3% which is within the range (25.1–41.0%) given by other authors [6, 7, 9, 19]. In our study cohort the median follow-up was 79 (range 12–148) months, and 10-year BRFS rate was 60% which is within the rage given by other authors [6, 20, 21]. Also median time to BR of 35 (range 8.00–86.0) months was similar as in other reports after RP [7, 9, 13] or after RT (68.9%) [20]. Our study indicated that GS and pT stage were not useful predictive factors for BRFS which may be in agreement with some studies [6] although in contrast with other [7]. This discrepancy in the statistical power of clinicopathological variables maybe caused by different inclusion criteria in other studies, analysis of bigger patients population with advanced and G4 tumours, too short follow-up or use of different levels for PSA BR (np. 0.1 ng/mL or 0.4 ng/mL instead of 0.2 ng/mL). However, the most important difference is caused by inclusion of tumour biological parameters to the multivariate analysis. Therefore, researchers are currently trying to understand (on molecular level) why some men with prostate cancer go on to develop aggressive disease with high risk of PCa death, whilst others maintain slow growing tumours [1].

Our study shows that increased PSA level was negative prognosticator for BR, as also shown in some studies [19–22] and its significance was not indicated in other [12, 18, 23]. Our ROC analysis showed the highest sensitivity (70–80%) for PSA, GLUT-1, and hTERT however, the highest specificity was indicated for Ki-67 (89.9%) and PSMA (61.4%). In our study, expression of proliferation marker - Ki-67 positively correlated with PSA suggesting that PSA, through androgen receptor, may result in promotion of PCa growth [24]. Ki-67 appeared to be negative prognostic factor for patients’ BRFS, but only in univariate analysis. In multivariate analysis however, we could not confirm its prognostic value which is in agreement with some authors results [9, 10] though in disagreement with other studies where prognostic value of Ki-67, among others, but not hypoxic biomarkers were indicated [7]. We suggest, after other authors, that hypoxia in PCa might have more predictive power than proliferation [6].

There is general agreement that various factors regulate angiogenesis in PCa. We studied VEGF expression to evaluate the angiogenic activity of PCa and have shown that a higher VEGF expression was a good prognosticator for patients’ BRFS which may suggest prognostic value of tumour remodeling and better oxygenation. Therefore, we assume, after other authors, that higher VEGF expression might be stimulated by cellular hypoxia [17] or androgens [25].

We did not observe the correlation between VEGF and the extent of hypoxia. However, in other study it was showed that androgens may activate hypoxia inducible factor -1 alph (HIF-1alpha) protein at the very early stage of prostate tumorigenesis which driving VEGF expression in androgen sensitive human prostate cancer [26]. In our study VEGF expression was not associated with tumour malignancy, tumour volume or pT stage which is in agreement with other studies [27]. Also MVD, as determined by CD34 antibody, was not correlated with tumour grade and pT stage and appeared to be non useful prognostic factor for patients’ BRFS as shown in some earlier study [23].

Tumour cells exist within a heterogenous tumour microenvironment with dynamic gradient of hypoxia that has been linked to malignant potential. This was shown in our study where GLUT-1 expression was associated with tumour grade and appeared to be independent negative prognosticator for BRFS. This finding supports earlier studies based on DNA signature [11], also those showing that hypoxic tumours fail to react to radiotherapy treatment and are at risk of BR [6]. Hypoxia also affects hTERT expression, the catalytic subunit of human telomerase, the lowest expression of which was observed in G3 tumours presenting the highest GLUT-1 expression. Our study demonstrated that higher nuclear expression of hTERT may be a good prognosticator for BRFS that may support finding that telomeres plays an important role in mitochondrial biology [28]. TERT under oxidative stress is reversibly excluded from the nucleus to mitochondria (nuclear-mitochondrial shuttling of TERT), where it is accumulated (lower nuclear TERT expression) and involved in the protective role in antioxidative stress, improvement of mitochondrial function, and better cell survival [28, 29]. Another explanation for our results is a hypothesis that telomerase activity is regulated by androgens and the wild-type human androgen receptor inhibits the expression of hTERT in the presence of androgen receptor agonists [30]. This may be true, as we observed it in our patients with anaplastic tumours, the highest PSA levels and the lowest nuclear hTERT expression [15].

Earlier we showed that higher PSMA expression in higher pTNM stages and tumour grades may indicate PSMA as a good marker of biological aggressiveness suitable for patients’ selection for more aggressive treatment [15]. However, now we are unable to show utility of PSMA for predicting patients’ prognosis for BRFS which is in agreement with some studies [22]. We could not indicate significant association between PSMA and tumour proliferation (Ki-67) which may be in line with other authors findings [22] and support the speculation that high expression of PSMA may be associated with unfavourable tumour phenotype such as hypoxia and be related with PCa development rather than progression [22]. Therefore, our data do not suggest a clinical utility of PSMA measurements for predicting patient prognosis. In our study, lack of significance of PSMA may be caused by relatively low number of analysed advanced PCa or the relatively low PSMA expression, which could be inhibited by a high level of pretreatment PSA. The negative relationship between PSMA and PSA was suggested by in vitro study. This may suggest that in patients with high testosterone or dihydrotestosterone level, low PSMA expression can be observed which may complicate easy application of PSMA/PET method, recommended recently for identification of localized recurrent PCa.

Our study supports a unique role of PCa tumour biology. Due to heterogeneity of PCa it is possible that a combination of biomarkers may provide better predictive value, which agrees with recent studies made on molecular level [11, 18]. However, these findings have to be confirmed by further analysis of prostate cancer-specific mortality or time to metastasis. Our study indicates the great role of hypoxia in BR induction in PCa. We first demonstrated that high pretreatment serum PSA level and high GLUT-1 expression or lower VEGF, and nuclear hTERT expression were negative predictors for 10-year BRFS rate in patients after RP. Therefore, we suggest to use these biomarkers for evaluating the patients’ prognosis. However, in our study PSA specificity for malignancy was only 56.2% and sensitivity - 73.3%. Therefore, it should be stressed that interval from PSA recurrence to metastasis is quite long and not every biochemical relapse leads to metastasis, because biochemical recurrences do not always form the most malignant neoplasms [21]. Therefore, only when PCa patients from high-risk group for BR develop metastases and have shorter OS, the prognostic significance of the biomarkers will be confirmed, and only then it will be possible to qualify patients to salvage therapy (for example with hypoxia-activated prodrugs), based on hypoxia biomarkers, instead of active surveillance.

## Conclusion

High pretreatment serum PSA and tumour hypoxia (high GLUT-1 expression or lower VEGF and lower nuclear hTERT expression) were negative prognosticators for biochemical recurrence-free survival in PCa patients after radical prostatectomy.
